# Trends and Age-Period-Cohort Effects on the Prevalence, Incidence and Mortality of Hepatocellular Carcinoma from 2008 to 2017 in Tianjin, China

**DOI:** 10.3390/ijerph18116034

**Published:** 2021-06-04

**Authors:** Chengyu Liu, Jing Wu, Zheng Chang

**Affiliations:** 1 School of Pharmaceutical Science and Technology, Tianjin University, Tianjin 300072, China; lcy0_0@163.com; 2 Center for Social Science Survey and Data, Tianjin University, Tianjin 300072, China; 3 Department of Medical Epidemiology and Biostatistics, Karolinska Institutet, 17177 Stockholm, Sweden; zheng.chang@ki.se

**Keywords:** hepatocellular carcinoma, prevalence, incidence, mortality, joinpoint regression analysis, age-period-cohort analysis

## Abstract

*Objectives:* China is the country most afflicted by hepatocellular carcinoma in the world. However, little is known about the epidemiology of hepatocellular carcinoma in China. This study aimed to examine the trends of the prevalence, incidence, and mortality of hepatocellular carcinoma in China, and to investigate the effects of age, period, and birth cohort on the epidemiological trend. *Methods:* The data were obtained from the Urban Employee Basic Medical Insurance claims database (2003–2017) in Tianjin, China, which covers 5.95 million individuals. The average annual percentage change of the prevalence, incidence, and mortality were accessed using joinpoint regression. Age-period-cohort models were produced to quantify the effects of age, period, and cohort. *Results:* The hepatocellular carcinoma prevalence rate increased by 5.13% annually from 20.12/100,000 in 2008 to 30.49/100,000 in 2017, and the incidence rate was almost unchanged, from 13.91/100,000 in 2008 to 14.09/100,000 in 2017, but mortality decreased by 1.80% annually from 8.18/100,000 in 2008 to 7.34/100,000 in 2017. The age-period-cohort analysis revealed that the prevalence rate was remarkably increased from age 25, peaked in age 60, and decreased at age 70 and over. In the period index, the prevalence rate increased gradually from 2008 to 2016, and decreased a little in 2017. In the cohort index, the prevalence rate decreased approximately linearly from the 1925 cohort to the 1990 cohort. The result for the incidence was similar to the prevalence. The mortality rate increased approximately linearly from age 45 to 85, decreased from the 1925 cohort to the 1990 cohort, but it changed a little with the change of period. *Conclusions:* The findings of this study could inform the necessity of conducting earlier screening for high-risk individuals and improving the treatment of hepatocellular carcinoma, which may also help to predict future changes in hepatocellular carcinoma epidemiology.

## 1. Introduction

Primary liver cancer is one of the most prevalent and deadly cancers worldwide, with about 905,667 new cases and 830,180 deaths occurring in 2020 [1]. China is the country most afflicted by liver cancer in the world, and about half of global newly diagnosed cases and deaths occur in China (410,038 new cases and 391,152 deaths in 2020) [1,2]. In patients with primary liver cancer, the major histological type is hepatocellular carcinoma (HCC), comprising about 90%, followed by intrahepatic cholangiocarcinoma and other rare types [3]. As HCC is responsible for a significant incidence and mortality around the world, resulting in a substantial economic burden, the description of the changing HCC epidemiological data is critical for the healthcare system.

Previous studies have reported worldwide or national trends on HCC epidemiology. Rich et al. suggested that the highest incidence of HCC in the world was in Asia and Africa, and the HCC incidence may have plateaued or begun to decrease in some Asian countries. Goh et al. also stated that Asia had the highest incidence of HCC worldwide (the age-standardized incidence rate of HCC in males in Eastern Asia was 31.9/100,000), and some Asian countries or regions had success in reducing HCC by introducing hepatitis B virus (HBV) vaccinations, such as Taiwan and Singapore [4]. Yeesoonsang et al. reported that the HCC incidence was expected to decrease among males and stabilize among females in the future [5]. Unlike in Asia, recent studies have reported that the HCC incidence has increased in low and medium incidence areas such as Western Europe and North America [6,7,8,9]. Wallace et al. reported that the age-adjusted incidence rate of HCC among Australian increased from 1.38/100,000 in 1982 to 4.96/100,000 in 2014, and White et al. revealed that the HCC incidence increased from 4.4/100,000 in 2000 to 6.7/100,000 in 2012 in the United States [7,9]. In addition, Bertuccio et al. reported that HCC mortality was observed to reduce in East Asia (e.g., Japan, Hong Kong and Korea) due to the control of HBV and hepatitis C virus (HCV) infections, but they still remained around 10–24/100,000 men and 2–8/100,000 women in Asia, which was 2- to 5-fold higher than those in most European and American countries [10].

However, there is no data on the epidemiology of HCC in mainland China, while these have been widely reported in the United States, Australia, Denmark, Thailand and many other counties and areas [4,5,6,7,8,9,10]. Only a few studies have reported the incidence and mortality rates of primary liver cancer in some regions of China, including Shanghai, Guangzhou, Shenzhen, Chongqing, Fuzhou, Nantong, Sihui and other cities [11,12,13,14,15,16,17,18,19,20,21]. However, almost all of the cities mentioned above are located in the south of China; the changes in prevalence, incidence, and mortality of primary liver cancer have not yet been examined in Northern China, and much less for HCC. In addition, the diet and lifestyle for people living in different countries or areas are different, and people in Northern China prefer to drink alcohol, which is an identified risk factor for HCC [22]. Therefore, it is essential to examine the epidemiology of HCC in mainland China, especially in Northern China.

Tianjin is one of the four municipalities in China, which is also the largest coastal opening city located in Northern China, ranking the seventh among the total 31 provinces/municipalities in terms of the gross domestic product per capita in mainland China. With the development of society, the living conditions, diet, and lifestyle, as well as the level of disease diagnosis and treatment have changed significantly, which may all affect the epidemiology of HCC. These factors in Tianjin also reflect the changes in many other cities in Northern China. This study aimed to describe the trends in HCC prevalence, incidence, and mortality rates over the past decade in Tianjin, Northern China, and to investigate the effects of age, period, and birth cohort on HCC prevalence, incidence, and mortality.

## 2. Method

### 2.1. Data Source

This population-based study was conducted using data obtained from the Urban Employee Basic Medical Insurance (UEBMI) claims database (2003–2017) in Tianjin, China. China has almost achieved the universal coverage of medical insurance through two systems: UEBMI and Urban and Rural Resident Basic Medical Insurance (URRBMI), which covers 1354.36 million inhabitants, accounting for 96.7% of the total Chinese population in 2019 [23]. The URRBMI program covers children, students and other unemployed adult residents living in urban and rural areas, and UEBMI enrollees represent all adult (at least 18 years) employees and retirees of the public and private sectors. Due to the different reimbursement benefits between the two types of medical insurance systems (the URRBMI program covers fewer healthcare items and pays less than UEBMI), inhabitants who are enrolled in the URRBMI program always have a lower utilization rate of healthcare resources than those enrolled in the UEBMI plan [24]. The evidence from the UEBMI claims database could more truly reflect the level of diagnosis and treatment, as well as the epidemiology data of a city. In Tianjin, there were about 11.37 million enrollees (UEBMI: 5.95 million; URRBMI: 5.42 million) in 2019, of which UEBMI enrollees accounted for over half [25]. The analytical sample in this study was thirty percent of the UEBMI enrollees randomly sampled based on their unique identification number. The UEBMI database consisted of inpatient, outpatient, and pharmacy service claims (the database from 2003 to 2007 included only the inpatient claims). The enrollment history, patient demographics (age, sex, working status), dates of service, diagnoses, medical prescription, and procedure information, as well as the related costs, were included in this database. Both the International Statistical Classification of Diseases and Related Health Problems 10th Revision (ICD-10) codes and medical records were used to identify the disease diagnoses. In addition, the all-cause death information was included in a separate dataset, which could be linked by patients’ unique identification number. This study was exempted from the application for ethical approval by the Safety and Ethics Committee of the School of Pharmaceutical Science and Technology in Tianjin University.

### 2.2. Target Population and Case Identification

The target population were the enrollees of UEBMI in the analytical sample during each calendar year from 2008 to 2017. The definition and identification of prevalent cases, incident cases and death cases are introduced as follows. The prevalent cases were patients with a diagnosis of HCC (ICD-10: C22.0 supplemented with Chinese descriptions) through the inpatient and outpatient claims, who were identified by the calendar year during 2008–2017 and included newly diagnosed patients and previously diagnosed cases. The incident cases (i.e., newly diagnosed patients) were identified from the prevalent cases. The year of the initial HCC diagnosis for each prevalent case was identified. The patients who had any diagnosis of HCC between 1 January 2003 and 31 December 2007 were excluded. Death cases (i.e., patients with HCC who died in each year) were also identified from the prevalent cases. The individual identification numbers were used to identify the death cases from a separate dataset mentioned above.

### 2.3. Statistical Analysis

The crude rate and age-standardized rate (ASR) were calculated. The crude prevalence, incidence, and mortality rate were defined as the number of prevalent cases, incident cases and death cases divided by the target population size, respectively. The crude rates were also calculated by sex (male/female), age groups (divided according to Segi’s World Standard Population, i.e., 20–24, 25–29, and 30–80 by 5 years, ≥85) and birth cohorts (1915–1919, 1920–1990 by 5 years). The ASR was calculated by summing up the products of the age-specific rates (*a_i_*, where *i* denotes the *i*th age class) and the number of persons (or weight, *w_i_*) in the same age subgroup *i* of the chosen standard population, then dividing the sum of the standard population [2] (or weights):

(1) $$\mathrm{ASR}=\frac{\sum_{i=1}^{A}a_{i}w_{i}}{\sum_{i=1}^{A}w_{i}}\times 100,000$$

Segi’s World Standard Population was used to standardize the prevalence, incidence, and mortality in this study [26].

#### 2.3.1. Joinpoint Regression Analysis

The joinpoint regression analysis was established to estimate the annual percent change (APC) for each segment and the average annual percent change (AAPC) over the entire period to quantify the trends of the age-standardized HCC prevalence, incidence, and mortality. This analysis was composed of a few continuous linear segments, which are always used to describe the trends in the outcome, and are also named as piecewise regression, segmented regression, broken line regression and multi-phase regression [27]. A general form of this model for observations ($x_{1},\;y_{1}),\;$($x_{2},\;y_{2}),\ldots \ldots ,(x_{n-1},\;y_{n-1}),\;\mathrm{and}\;\left(x_{n},\;y_{n}\right)\;$ could be written as follows:

(2) $$E\left[y|x\right]\;=\;\beta_{0}+\beta_{1}x+\delta_{1}{\left(x-\tau_{1}\right)}^{+}+\ldots \ldots +\delta_{k}{\left(x-\tau_{k}\right)}^{+}$$

where $\;\tau_{1},\;\tau_{2},\ldots \ldots ,\tau_{k-1},\;\tau_{k}$ are the unknown joinpoints and ${\left(x-\tau_{k}\right)}^{+}\;=\;\left(x-\tau_{k}\right)\;$ for $\left(x-\tau_{k}\right)\;>\;0,\;$ and 0 otherwise [27]. The analysis starts with the zero joinpoint, representing a straight line, and tests whether more joinpoints are statistically significant and should be added to the model. The significant joinpoints were identified by a Monte Carlo Permutation method [27]. In the final model, each joinpoint denotes a significant change in the trend of the line segment separated by this joinpoint. The annual percent change for each line segment and the corresponding 95% confidence intervals (CI) are reported in the final model.

#### 2.3.2. Age-Period-Cohort Analysis

The age-period-cohort analysis was performed to evaluate the net effects of age, period, and cohort on HCC prevalence, incidence, and mortality simultaneously. Both age (from 20 to 85 years old) and period (from 2008 to 2017) were subdivided by 1-year intervals, and the birth cohort was calculated by subtracting the age from the period. The age-period-cohort models in this study were based on a Poisson log-linear model with an intrinsic estimator (IE), which was widely used to avoid linear dependency (i.e., period = age + cohort) and to disentangle the three effects of age, period, and cohort [28]. The IE approaches the estimator of the age-period-cohort model by applying the estimable functions and the singular value decomposition of the matrices, and it generates the coefficients of the effects, which are exponentially expressed as rate ratios [28]. The model could be generally expressed as:

(3) $$log\left(Y_{j}\right)\;=\;\mu +\alpha \;age_{j}+\beta \;period_{j}+\gamma \;cohort_{j}+\varepsilon$$

where the Y denotes the HCC prevalence, incidence, and mortality of the corresponding age group *j*; the *α*, *β* and *γ* denote the corresponding age, period, and cohort effects; μ is the intercept item; and ε  is the random error. A full age-period-cohort model fitted better than any combination of age, period, and cohort factors (Supplementary Table S1).

The statistical analyses were performed using Joinpoint Regression Program V.4.7.0.0 and Stata V.13.0. The significant level was defined as two-sided alpha = 0.05.

## 3. Results

**Figure 1:**
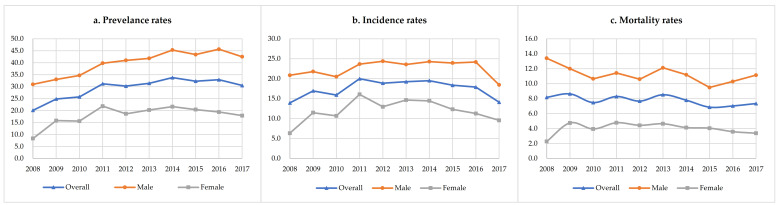
Trends of the age-standardized prevalence, incidence, and mortality rates of HCC from 2008 to 2017.

**Figure 2:**
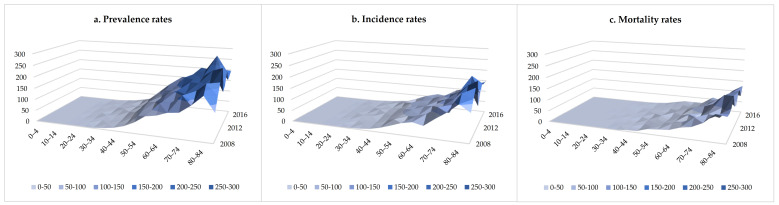
Age-specific prevalence, incidence, and mortality rates of HCC from 2008 to 2017.

**Figure 3:**
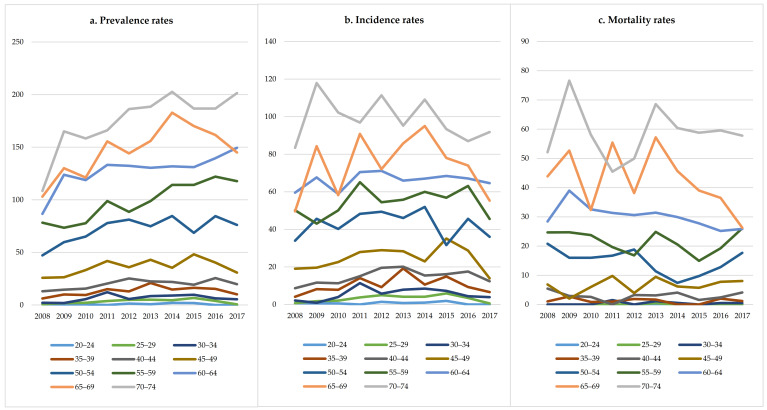
Temporal trends of the prevalence, incidence, and mortality rates of HCC by age group from 2008 to 2017. Note: As there was no apparent trend among individuals aged 75−79, 80−84, and ≥85, these three age groups were not shown to enhance the clarity and readability of this figure.

**Figure 4:**
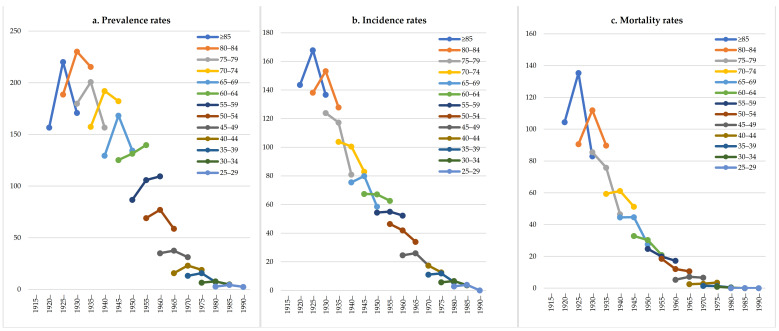
Cohort-specific prevalence, incidence, and mortality rates of HCC by age group.

Over the study period from 2008 to 2017, a total of 3811 men and 1834 women with HCC were identified, of which 3563 men and 1742 women were new cases, and 2163 men and 722 women died during the follow-up. The prevalence, incidence, and mortality of males were about twice those of females (Figure 1). The number of patients with HCC increased from 447 (crude prevalence rate: 38.07/100,000; ASR: 20.12/100,000) in 2008 to 1141 (crude prevalence rate: 71.37/100,000; ASR: 30.49/100,000) in 2017. There were 334 new cases (crude incidence rate: 26.65/100,000; ASR: 13.91/100,000) in 2008 and 524 new cases (crude incidence rate: 32.78/100,000; ASR: 14.09/100,000) in 2017. The number of deaths increased from 196 (crude mortality rate: 15.64/100,000; ASR: 8.18/100,000) in 2008 to 281 (crude mortality rate: 17.58/100,000; ASR: 7.34/100,000) in 2017 (Table 1). Figure 2 shows the age-specific prevalence, incidence, and mortality rates of HCC from 2008 to 2017. The prevalence rate of HCC was lower among individuals under the age of 30 (<5.0/100,000; average value between 2008 and 2017, and the same below); it increased for the 30–34 age group (6.6/100,000) and peaked in 80–84 age group (217.1/100,000). The incidence rate of HCC also increased from 30–34 age group (5.6/100,000) and peaked in 80–84 age group (156.2/100,000). The mortality rate of HCC was lower under the age of 45 (<5.0/100,000), and continuously increased from the 45–49 age group (6.6/100,000) to the ≥85 age group (114.6/100,000). Figure 3 showed the temporal trends of the prevalence, incidence, and mortality rates of HCC by age group from 2008 to 2017. The prevalence rate of HCC among individuals aged 50–54, 55–59, 60–64, 65–69 and 70–74 was found to increase from 2008 to 2017, but there was no apparent trend in any of the other age groups. The incidence and mortality rates of HCC among the different age groups had no obvious temporal trends from 2008 to 2017. The cohort-specific prevalence, incidence, and mortality rates of HCC by age group are shown in Figure 4. While the prevalence rate among those aged 55–59 and 60–64 was found to increase steadily with each successive birth cohort, there was no apparent trend in any of the other age groups. The incidence rates in the different age groups all decreased with the birth cohort even though there were some fluctuations in several groups. The mortality rate in the different age groups almost all decreased with the birth cohort, except for 40–44 and 45–49.

Table 2 shows the results of the joinpoint regression analysis. The prevalence rates among the overall patients, men, and women all increased from 2008 to 2017, but the changes for the women were not statistically significant (overall: AAPC: 5.13%; 95% CI: 1.56%–8.83%; *p* = 0.005. Men: AAPC: 3.62%; 95% CI: 1.54%–5.76%; *p* = 0.001. Women: AAPC: 7.01%; 95% CI: −1.52–16.27%; *p* = 0.110). There was no remarkable trend in the HCC incidence rates among the overall patients (AAPC: −0.82%; 95% CI: −3.62%–2.07%; *p* = 0.576), men (AAPC: 3.98%; 95% CI: −5.6%–14.53%; *p* = 0.429) or women (AAPC: −0.1%; 95% CI: −5.85%–5.99%; *p* = 0.969) from 2008 to 2015, but a significant increasing trend was found in the incidence rate among men during 2008–2015 (APC: 2.71 %; 95% CI: 0.48%–4.98%; *p* = 0.026). The mortality rates among the overall patients and women significantly decreased from 2008 to 2017 with the AAPC of −1.8% (95% CI: −3.37%–−0.2%; *p* = 0.032) and −12.19% (95% CI: −22.32%–−0.75%; *p* = 0.040), respectively, and the mortality rate among men remained stable (AAPC: −0.1%; 95% CI: −5.85%–5.99%; *p* = 0.969).

**Figure 5:**
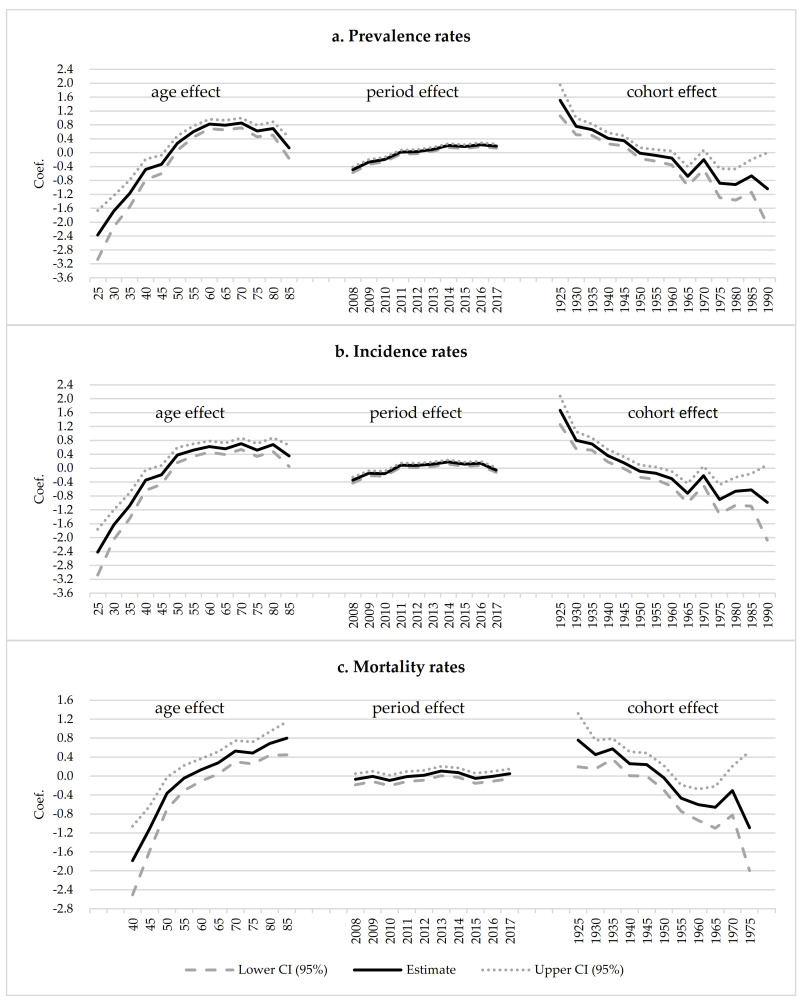
The age, period, and cohort effects on the prevalence, incidence, and mortality of HCC. Note: Some categories of the age and cohort factors are not shown in the figure due to the lack of space. Please refer to Supplementary Table S2 for the detailed statistics.

Figure 5 and Supplementary Tables S2–S4 reflect the effects of age, period, and birth cohort on the prevalence, incidence, and mortality of HCC. The prevalence rate of HCC was remarkably increased from age 25, peaked in age 60, and decreased at age 70 and over. The prevalence rate for people aged 70 years was 24.2 times higher than that for people at 25 years old. In the period index, the prevalence rate increased slightly from 2008 to 2017, which in 2017 was twice as many as 2008. In the cohort index, the prevalence rate decreased approximately linearly from 1925 cohort to 1990 cohort, and that for people born in 1920 was 12.7 times as many as in 1990. The tendencies of the age, period, and birth cohort effects on the incidence rate were similar to the prevalence rate. The incidence rate for people aged 70 years was 21.8 times higher than that for people at 25 years old, and it was 14.2 times as many as those born in 1920 for people born in 1990. The incidence rate in 2017 was 1.3 times as many as in 2008. There were smaller age, period, and birth cohort effects on mortality than the prevalence and incidence. The mortality rate of HCC increased from age 40 to 85, which was 12.2 times higher for people at 85 years old than for people aged 40 years. However, the period had no significant effect on the mortality rate. The tendency of the birth cohort’s effect on the mortality rate was similar to incidence and prevalence rate, and the mortality rate for people born in 1920 was 6.3 times as many as that for people born in 1970.

## 4. Discussion

This is the first study to reveal the prevalence, incidence, and mortality rates of HCC and the effects of age, period, and birth cohort on them in Northern China, taking Tianjin as an example. In addition, this is also the first study to report the prevalence, incidence, and mortality of HCC in mainland China. This population-based study revealed that the age-standardized incidence rate of HCC in Tianjin ranged between 13.91 and 19.98/100,000 during 2008–2017, which was substantially higher than that in other countries [7,8,9,29]. The age-standardized mortality rate of HCC based on the world standard population among men in Tianjin was much higher than that in Europe and America, such as France, Germany, United Kingdom, United States, and Australia, but lower than Japan, Hong Kong, and Republic of Korea [10]. The age-standardized mortality rate among women in Tianjin was much higher than that in France, Germany, United Kingdom, United States, Australia, Japan, Hong Kong and many of the other countries or areas, but lower than Republic of Korea [10]. The population in this study were employees and retirees derived from the UEBMI database in Tianjin who had stronger awareness of health and had easier access to better medical care than inhabitants who were enrolled in another basic medical insurance (i.e., URRBMI), which may partly explain why the mortality rate in this study was lower than that in Japan, Hong Kong, and the Republic of Korea. In addition, the incidence, and mortality of HCC among men are always much higher than those among women, which could be explained by some environmental exposures with differential risks between the sexes, e.g., heavy alcohol use.

Because most of the published studies reported the prevalence, incidence, and mortality rate for liver cancer in China but did not focus on HCC, we compared the time trends on the prevalence, incidence, and mortality of HCC in Tianjin with those of liver cancer in other areas in mainland China over the past decade. The prevalence rate in this study showed a significant increasing trend from 2008 to 2017, which was similar to the trend for liver cancer in China [30]. The incidence rate of HCC in Tianjin was almost unchanged over the past decade, and it was similar to Chongqing [21]. However, there were other studies that reported that the incidence of liver cancer was decreasing in China, Shenzhen city and Taizhou city [17,19,31]. The mortality rate of HCC in this study showed a decreased trend during 2008–2017, and this was similar to the trend of liver cancer in China as well as and Tieling city [12,14,31].

The age-period-cohort analysis results suggested that age exhibited a strong association with HCC prevalence, incidence, and mortality in Tianjin. Age is always considered as the main factor in age-period-cohort analysis, because it could represent consistent extrinsic factors, such as the accretion of mutations and accumulative exposures to carcinogens over time, which would increase the risk of developing cancers [32]. As is known to us, the elderly also have a higher risk of death than the young. To be mentioned in this study, the incidence rate increased substantially with age before 50 years old, and then flattened, which meant that the incidence rate of the population aged 50–80 was the highest in all age groups. The range of ages with the highest incidence in this study is wider than that which was reported in the United States [33]. Liu et al. reported that the incidence of HCC increased from ages 25–29 years before peaking at ages 50–54 years and declined thereafter in the United States [33]. The high-incidence age in this study was also much earlier than that in other countries [5,7]. The earlier typical onset age and the larger proportion of elderly HCC patients result in the huge economic burden of HCC in China. In addition, it is conceivable that the number of high-risk individuals in the future will increase with China’s rapidly aging population. Therefore, earlier screening for high-risk individuals could be conducted to prevent HCC.

The period effect is usually affected by socioeconomic development and historical events, including public health emergencies (e.g., economic crises, epidemics of infectious diseases), public health interventions (e.g., disease screening, detection levels), lifestyle behaviors in different periods, wars, and other factors. With the accelerating pace of life and work, lifestyle has changed over the past decade. More and more people are suffering from increasing mental stress, staying up late, drinking, obesity, and long-term fatigue, which may do damage to the liver. The impact of unhealthy lifestyles and environmental degradation may be the reason why the prevalence and incidence rate of HCC in 2017 were 1.5–2 times as many as those in 2008. In addition, there was no period effect on the mortality of HCC, which meant that the treatment of HCC was not improved from 2008 to 2017. Because this study only examined the ten-year trend, an analysis based on longer-term data may reflect a more remarkable period effect on the epidemiology of HCC.

Cohort effects represent variations of some characteristics over time among groups of individuals who were born in the same year or decade or are defined by other shared experience. The cohort effect obtained by the age-period-cohort model was the net effect related to birth year after deducting the impact of age and period effects. The HCC prevalence, incidence, and mortality for individuals born in recent years were remarkably lower than the earlier cohorts in this study. The probable reasons were the effective control of HBV and HCV infection attributed to the increasingly wide availability of HBV vaccines and the use of new antiviral drugs for HBV and HCV, and the lower infection risk of aflatoxins, as well as the stronger awareness of health and disease prevention among the later birth cohorts than the earlier birth cohorts. There were two fluctuations around 1935 and 1960 which reflected the influence of the Japanese invasion of China and the social and economic system changes on inhabitants’ health. This is similar to the previous study [34].

This study was subject to several limitations. First, the cases identified from the claim database are “prevalent-treatment cases”, and the number may be lower than the true number of prevalent cases. However, this underestimation would not affect the analyses of the time trends on the prevalence rates. Second, because the database used in this study was the claims for employees and retirees, the population under the age of 18 was not included, which may underestimate the prevalence and incidence as well as overestimating the mortality. However, a recent study reported that the prevalence was under 1.00/100,000 for individuals under 20 years old [30], which may have a very small effect on the prevalence of the total population. Third, this study was conducted only on the UEBMI database in Tianjin; the study population may not represent patients with different types of insurance or no insurance coverage. However, the diagnosis of HCC is unlikely to differ by insurance status; thus, the results in this study may apply to other cities with similar levels of economy to Tianjin in Northern China. Fourth, this study only reported the trend in the prevalence, incidence, and mortality of HCC from 2008 to 2017, as it was limited by the current database. As Sorafenib was covered by the National Reimbursement Drug List in 2017 and Lenvatinib was approved by the National Medical Products Administration in 2018 in China, a future study focused on longer-term trends could be conducted to examine the impact of new interventions on the mortality of HCC patients. Lastly, as there is no epidemiological study focused on HCC in China, we compared the time trend in the prevalence, incidence, and mortality of HCC with those of liver cancer.

## 5. Conclusions

In the past decade, HCC incidence has changed a little and mortality has decreased, so the prevalence has shown an increasing trend in Tianjin, China. The strong age and cohort effects and a small period effect on the prevalence, incidence, and mortality were found. The findings of this study could inform the necessity of conducting earlier screening for high-risk individuals and improving the treatment of HCC, which may also help to predict future changes in HCC epidemiology.

## Figures and Tables

**Table 1:** Estimated prevalence, incidence, and mortality of HCC from 2008 to 2017.

Year	Overall			Male			Female		
	Cases	Crude Rate (1/10^5^)	ASR ^†^ (1/10^5^)	Cases	Crude Rate (1/10^5^)	ASR ^†^ (1/10^5^)	Cases	Crude Rate (1/10^5^)	ASR ^†^ (1/10^5^)
**Prevalence**									
2008	477	38.07	20.12	386	60.22	30.97	91	14.87	8.34
2009	649	46.22	24.85	463	62.81	33.01	186	27.88	15.80
2010	715	46.86	25.73	519	63.56	34.67	196	27.64	15.60
2011	874	60.36	31.19	586	76.65	39.78	288	42.13	21.85
2012	894	58.68	30.24	632	78.84	40.98	262	36.30	18.66
2013	982	61.40	31.41	684	81.19	41.82	298	39.37	20.23
2014	1075	70.33	33.76	743	93.16	45.31	332	45.42	21.65
2015	1102	65.45	32.28	763	86.11	43.41	339	42.51	20.42
2016	1176	72.00	32.88	827	96.80	45.64	349	44.80	19.40
2017	1141	71.37	30.49	809	97.30	42.50	332	43.27	17.89
**Incidence**									
2008	334	26.65	13.91	264	41.19	20.86	70	11.43	6.33
2009	443	31.55	16.93	305	41.38	21.78	138	20.69	11.48
2010	445	29.17	15.91	310	37.96	20.49	135	19.04	10.67
2011	557	38.47	19.98	346	45.26	23.66	211	30.87	16.06
2012	557	36.56	18.87	375	46.78	24.37	182	25.21	12.96
2013	592	37.01	19.24	379	44.99	23.55	213	28.14	14.65
2014	612	40.04	19.50	393	49.28	24.28	219	29.96	14.43
2015	611	36.29	18.36	410	46.27	23.94	201	25.20	12.32
2016	630	38.57	17.88	430	50.33	24.16	200	25.67	11.27
2017	524	32.78	14.09	351	42.21	18.43	173	22.55	9.56
**Mortality ^‡^**									
2008	196	15.64	8.18	171	26.68	13.40	25	4.08	2.28
2009	226	16.09	8.63	172	23.33	12.00	54	8.09	4.75
2010	213	13.96	7.47	165	20.21	10.65	48	6.77	3.94
2011	235	16.23	8.28	173	22.63	11.42	62	9.07	4.77
2012	240	15.75	7.67	175	21.83	10.60	65	9.00	4.43
2013	272	17.01	8.52	202	23.98	12.12	70	9.25	4.63
2014	253	16.55	7.79	188	23.57	11.19	65	8.89	4.12
2015	246	14.61	6.86	179	20.20	9.49	67	8.40	4.04
2016	261	15.98	7.02	194	22.71	10.29	67	8.60	3.58
2017	281	17.58	7.34	216	25.98	11.14	65	8.47	3.37

^†^ASR—age-standardized rate, and Segi’s world population was used for ASR. ^‡^ The death cases used to calculate the mortality rate in a particular year were defined as the patients who had claims related to HCC in that year, and they do not include the patients with a diagnosis of HCC in the early year but who did not see a doctor in that year.

**Table 2:** Results of the joinpoint regression on the age-adjusted prevalence, incidence, and mortality rates of the HCC from 2008 to 2017.

Group	Joinpoint	Years	APC (95%CI)	*p*-Value	AAPC (95%CI)	*p*-Value
**Prevalence**						
Overall	1	2008–2011	14.97 (1.83, 29.81)	0.032 *	5.13 (1.56, 8.83)	0.005 *
		2011–2017	0.53 (−2.52, 3.68)	0.678		
Male	1	2008–2014	6.38 (3.69, 9.14)	0.002 *	3.62 (1.54, 5.76)	0.001 *
		2014–2017	−1.68 (−7.55, 4.57)	0.512		
Female	1	2008–2011	27.65 (−5.29, 72.05)	0.090	7.01 (−1.52, 16.27)	0.110
		2011–2017	−2.03 (−8.32, 4.70)	0.463		
**Incidence**						
Overall	1	2008–2014	5.05 (−0.47, 10.88)	0.066	−0.31 (−4.77, 4.35)	0.893
		2014–2017	−10.24 (−22.27, 3.66)	0.112		
Male	1	2008–2015	2.71 (0.48, 4.98)	0.026 *	−0.82 (−3.62, 2.07)	0.576
		2015–2017	−12.22 (−24.51, 2.07)	0.077		
Female	1	2008–2011	29.10 (−8.30, 81.75)	0.113	3.98 (−5.60, 14.53)	0.429
		2011–2017	−6.69 (−14.13, 1.41)	0.085		
**Mortality**						
Overall	0	2008–2017	−1.80 (−3.37, −0.20)	0.032 *	−1.80 (−3.37, −0.20)	0.032 *
Male	0	2008–2017	−0.10 (−5.85, 5.99)	0.969	−0.10 (−5.85, 5.99)	0.969
Female	0	2008–2017	−12.19 (−22.32, −0.75)	0.040 *	−12.19 (−22.32, −0.75)	0.040 *

* *p* < 0.05. APC: annual percentage change; AAPC: average annual percentage change; 95% CI: 95% confidence interval.

## Data Availability

The data that support the findings of this study were available from the Tianjin Healthcare Security Administration. Due to the requirement from the data owner, these data could only be used for this study under the license, which could not be shared to others.

## Supplementary Materials

The following are available online at https://www.mdpi.com/article/10.3390/ijerph18116034/s1. Table S1: Goodness of fit statistics for the combination of age, period and cohort factors. Table S2: Results of the age-period-cohort analysis on the prevalence of HCC. Table S3: Results of the age-period-cohort analysis on the incidence of HCC. Table S4: Results of the age-period-cohort analysis on the mortality of HCC.
