# Supplementary material for: Trends and Age-Period-Cohort Effects on the Prevalence, Incidence and Mortality of Hepatocellular Carcinoma from 2008 to 2017 in Tianjin, China
Source: Int J Environ Res Public Health. 2021 Jun 4;18(11):6034. doi: 10.3390/ijerph18116034 (PMC8200005; doi:10.3390/ijerph18116034)
Supplement: Supplementary file 1 [file ijerph-18-06034-s001.zip › ijerph-1221623-supplementary.pdf]

Supplementary Materials:

Table S1 Goodness of fit statistics for the combination of age: period, cohort factors.

	Model	d.f.	Deviance	AIC
	Age	549	744.9	3277.0
	Period	600	8855.0	11285.0
	Cohort	540	1231.7	3781.8
**Prevalence rate**	Age+period	540	596.6	3146.7
	Age+cohort	480	514.4	3184.5
	Period+cohort	531	605.4	3173.5
	**Age+period+cohort**	**472**	**452.4**	**3138.4**
	Age	549	648.6	2919.1
	Period	600	4773.9	6942.5
	Cohort	540	784.1	3072.7
**Incidence rate**	Age+period	540	586.9	2875.4
	Age+cohort	480	580.2	2988.7
	Period+cohort	531	601.8	2908.4
	**Age+period+cohort**	**472**	**513.1**	**2837.7**
	Age	414	476.4	2000.2
	Period	450	2136.7	3588.6
	Cohort	405	509.3	2051.1
**Mortality rate**	Age+period	405	460.4	2002.2
	Age+cohort	360	424.6	2056.4
	Period+cohort	396	460.3	2020.1
	**Age+period+cohort**	**352**	**418.4**	**2026.2**

d.f., degree of freedom; AIC, Akaike information criterion, are calculated by R software; the smaller the AIC and Deviance, the better the model fit. The best fitting models for each dataset are highlighted in bold.

Table S2. The results of age-period-cohort analysis on prevalence of HCC.

	Coef.	Std. Err.	z	P>z	[95% Conf.Interval]	
**age**						
25	-2.369	0.358	-6.62	0.000	-3.070	-1.668
26	-2.167	0.305	-7.1	0.000	-2.766	-1.569
27	-1.982	0.272	-7.3	0.000	-2.514	-1.450
28	-1.849	0.253	-7.3	0.000	-2.345	-1.352
29	-1.393	0.209	-6.66	0.000	-1.803	-0.983
30	-1.683	0.229	-7.36	0.000	-2.131	-1.235
35	-1.175	0.196	-6	0.000	-1.558	-0.791
36	-1.198	0.199	-6.02	0.000	-1.589	-0.808
37	-0.667	0.162	-4.11	0.000	-0.986	-0.349
38	-0.766	0.165	-4.64	0.000	-1.089	-0.442
39	-0.657	0.159	-4.12	0.000	-0.970	-0.345
40	-0.479	0.150	-3.19	0.001	-0.774	-0.185
45	-0.335	0.136	-2.46	0.014	-0.602	-0.069
50	0.277	0.105	2.64	0.008	0.071	0.483
55	0.610	0.083	7.33	0.000	0.447	0.774
60	0.827	0.071	11.63	0.000	0.687	0.966
65	0.794	0.070	11.28	0.000	0.656	0.931
70	0.857	0.072	11.88	0.000	0.716	0.999
75	0.626	0.084	7.41	0.000	0.460	0.791
80	0.695	0.098	7.1	0.000	0.503	0.887
85	0.137	0.156	0.88	0.380	-0.169	0.443
**period**						
2008	-0.489	0.041	-11.92	0.000	-0.570	-0.409
2009	-0.267	0.035	-7.64	0.000	-0.336	-0.199
2010	-0.196	0.033	-6	0.000	-0.260	-0.132
2011	0.017	0.029	0.58	0.561	-0.040	0.075
2012	0.032	0.029	1.12	0.262	-0.024	0.088
2013	0.096	0.028	3.49	0.000	0.042	0.150
2014	0.208	0.027	7.72	0.000	0.155	0.261
2015	0.176	0.027	6.43	0.000	0.123	0.230
2016	0.231	0.028	8.32	0.000	0.176	0.285
2017	0.192	0.029	6.54	0.000	0.134	0.249
**birth cohort**						
1925	1.508	0.227	6.65	0.000	1.064	1.952
1926	1.184	0.213	5.57	0.000	0.767	1.601
1927	0.878	0.191	4.6	0.000	0.504	1.252
1928	1.142	0.142	8.03	0.000	0.863	1.420
1929	0.696	0.146	4.77	0.000	0.410	0.982

1930	0.760	0.123	6.2	0.000	0.520	1.001
1935	0.663	0.084	7.89	0.000	0.499	0.828
1940	0.416	0.082	5.05	0.000	0.254	0.577
1945	0.342	0.074	4.64	0.000	0.198	0.487
1950	-0.011	0.079	-0.14	0.890	-0.167	0.145
1955	-0.079	0.084	-0.94	0.347	-0.245	0.086
1960	-0.152	0.102	-1.49	0.136	-0.352	0.048
1965	-0.679	0.142	-4.79	0.000	-0.957	-0.402
1970	-0.201	0.141	-1.42	0.155	-0.478	0.076
1975	-0.881	0.212	-4.15	0.000	-1.296	-0.465
1980	-0.918	0.228	-4.02	0.000	-1.365	-0.471
1985	-0.668	0.241	-2.77	0.006	-1.141	-0.196
1990	-1.035	0.528	-1.96	0.050	-2.070	0.001
_cons	-7.598	0.029	-263.65	0.000	-7.654	-7.541

Table S3. The results of age-period-cohort analysis on incidence of HCC.

	**Coef.**	**Std. Err.**	**z**	**P>z**	**[95% Conf.Interval]**	
**age**						
25	-2.419	0.336	-7.2	0.000	-3.077	-1.761
26	-2.021	0.270	-7.49	0.000	-2.550	-1.492
27	-1.885	0.248	-7.6	0.000	-2.372	-1.399
28	-1.814	0.238	-7.64	0.000	-2.280	-1.349
29	-1.287	0.193	-6.68	0.000	-1.665	-0.909
30	-1.635	0.218	-7.51	0.000	-2.062	-1.209
35	-1.081	0.190	-5.68	0.000	-1.454	-0.708
36	-1.005	0.186	-5.39	0.000	-1.370	-0.639
37	-0.513	0.156	-3.29	0.001	-0.818	-0.208
38	-0.729	0.168	-4.34	0.000	-1.058	-0.399
39	-0.497	0.156	-3.18	0.001	-0.803	-0.191
40	-0.345	0.149	-2.31	0.021	-0.637	-0.052
45	-0.193	0.139	-1.39	0.164	-0.466	0.079
50	0.377	0.110	3.42	0.001	0.161	0.592
55	0.518	0.092	5.63	0.000	0.337	0.698
60	0.619	0.082	7.54	0.000	0.458	0.780
65	0.557	0.086	6.51	0.000	0.389	0.725
70	0.707	0.085	8.33	0.000	0.540	0.873
75	0.520	0.093	5.58	0.000	0.337	0.702
80	0.680	0.102	6.67	0.000	0.480	0.879
85	0.355	0.154	2.3	0.021	0.053	0.658
**period**						

2008	-0.345	0.042	-8.13	0.000	-0.428	-0.262
2009	-0.145	0.037	-3.97	0.000	-0.217	-0.074
2010	-0.162	0.036	-4.52	0.000	-0.232	-0.092
2011	0.086	0.032	2.7	0.007	0.024	0.149
2012	0.078	0.032	2.47	0.013	0.016	0.141
2013	0.114	0.031	3.69	0.000	0.053	0.175
2014	0.176	0.031	5.73	0.000	0.116	0.237
2015	0.114	0.031	3.62	0.000	0.052	0.176
2016	0.138	0.032	4.36	0.000	0.076	0.200
2017	-0.055	0.035	-1.57	0.116	-0.123	0.013
**birth cohort**						
1925	1.662	0.209	7.96	0.000	1.253	2.072
1926	1.003	0.227	4.43	0.000	0.559	1.448
1927	0.828	0.195	4.24	0.000	0.445	1.210
1928	0.985	0.152	6.49	0.000	0.688	1.282
1929	0.818	0.143	5.73	0.000	0.538	1.098
1930	0.802	0.125	6.42	0.000	0.557	1.047
1935	0.695	0.089	7.81	0.000	0.521	0.870
1940	0.358	0.092	3.89	0.000	0.177	0.538
1945	0.155	0.087	1.79	0.074	-0.015	0.326
1950	-0.087	0.090	-0.96	0.338	-0.264	0.090
1955	-0.146	0.092	-1.59	0.112	-0.327	0.034
1960	-0.303	0.109	-2.79	0.005	-0.516	-0.090
1965	-0.722	0.144	-5.02	0.000	-1.005	-0.440
1970	-0.219	0.140	-1.56	0.119	-0.494	0.056
1975	-0.900	0.215	-4.18	0.000	-1.321	-0.478
1980	-0.664	0.206	-3.22	0.001	-1.068	-0.260
1985	-0.626	0.239	-2.62	0.009	-1.094	-0.157
1990	-0.988	0.553	-1.79	0.074	-2.072	0.095
_cons	-7.983	0.027	-294.43	0.000	-8.036	-7.930

Table S4. The results of age-period-cohort analysis on mortality of HCC.

	Coef.	Std. Err.	z	P>z	[95% Conf.Interval]	
**age**						
40	-1.783	0.370	-4.820	0.000	-2.508	-1.058
45	-1.107	0.241	-4.590	0.000	-1.580	-0.635
50	-0.363	0.172	-2.110	0.035	-0.699	-0.026
55	-0.043	0.136	-0.320	0.752	-0.308	0.223
60	0.131	0.122	1.080	0.280	-0.107	0.370
65	0.279	0.119	2.340	0.019	0.045	0.512

70	0.526	0.114	4.620	0.000	0.302	0.749
75	0.486	0.118	4.110	0.000	0.254	0.717
80	0.686	0.126	5.440	0.000	0.439	0.934
85	0.797	0.179	4.460	0.000	0.447	1.147
**period**						
2008	-0.070	0.060	-1.180	0.239	-0.187	0.047
2009	-0.010	0.055	-0.170	0.862	-0.118	0.099
2010	-0.093	0.056	-1.670	0.094	-0.203	0.016
2011	-0.011	0.054	-0.200	0.838	-0.116	0.094
2012	0.014	0.053	0.270	0.786	-0.089	0.117
2013	0.105	0.050	2.110	0.035	0.007	0.203
2014	0.072	0.051	1.400	0.160	-0.029	0.173
2015	-0.048	0.053	-0.890	0.371	-0.152	0.057
2016	-0.007	0.052	-0.140	0.892	-0.109	0.095
2017	0.047	0.052	0.920	0.360	-0.054	0.148
**birth cohort**						
1925	0.758	0.287	2.640	0.008	0.194	1.321
1926	0.557	0.270	2.060	0.039	0.028	1.086
1927	0.314	0.246	1.270	0.203	-0.169	0.796
1928	0.497	0.192	2.590	0.010	0.121	0.873
1929	0.575	0.166	3.460	0.001	0.249	0.900
1930	0.452	0.154	2.940	0.003	0.151	0.753
1935	0.574	0.110	5.190	0.000	0.357	0.790
1940	0.260	0.130	2.000	0.046	0.005	0.514
1945	0.242	0.124	1.940	0.052	-0.002	0.485
1950	-0.037	0.131	-0.280	0.780	-0.294	0.221
1955	-0.466	0.141	-3.310	0.001	-0.742	-0.190
1960	-0.604	0.169	-3.570	0.000	-0.935	-0.272
1965	-0.659	0.225	-2.930	0.003	-1.100	-0.217
1970	-0.309	0.263	-1.180	0.240	-0.825	0.206
1975	-1.090	0.820	-1.330	0.184	-1.997	0.517
_cons	-8.273	0.040	-207.140	0.000	-8.351	-8.194
